# Pharmacokinetics of intravenous pan-class I phosphatidylinositol 3-kinase (PI3K) inhibitor [^14^C]copanlisib (BAY 80-6946) in a mass balance study in healthy male volunteers

**DOI:** 10.1007/s00280-017-3383-9

**Published:** 2017-07-11

**Authors:** Michael Gerisch, Thomas Schwarz, Dieter Lang, Gabriele Rohde, Stefanie Reif, Isabelle Genvresse, Susanne Reschke, Dorina van der Mey, Camille Granvil

**Affiliations:** 1 Bayer Aktiengesellschaft, DMPK, 42096 Wuppertal, Germany; 2 Bayer Aktiengesellschaft, Clinical Sciences, Berlin, Germany; 3 Bayer Aktiengesellschaft, Clinical Sciences, Wuppertal, Germany; 4 Bayer HealthCare Pharmaceuticals Inc., Whippany, USA

**Keywords:** Copanlisib, BAY 80-9646, Mass balance, ADME, Pharmacokinetics, Human

## Abstract

**Purpose:**

To determine the pharmacokinetics of radiolabeled copanlisib (BAY 80-6946) in healthy male volunteers and to investigate the disposition and biotransformation of copanlisib.

**Methods:**

A single dose of 12 mg copanlisib containing 2.76 MBq [^14^C]copanlisib was administered as a 1-h intravenous infusion to 6 volunteers with subsequent sampling up to 34 days. Blood, plasma, urine and feces were collected to monitor total radioactivity, parent compound and metabolites.

**Results:**

Copanlisib treatment was well tolerated. Copanlisib was rapidly distributed throughout the body with a volume distribution of 1870 L and an elimination half-life of 52.1-h (range 40.4–67.5-h). Copanlisib was the predominant component in human plasma (84% of total radioactivity AUC) and the morpholinone metabolite M1 was the only circulating metabolite (about 5%). Excretion of drug-derived radioactivity based on all 6 subjects was 86% of the dose within a collection interval of 20–34 days with 64% excreted into feces as major route of elimination and 22% into urine. Unchanged copanlisib was the main component excreted into urine (15% of dose) and feces (30% of dose). Excreted metabolites (41% of dose) of copanlisib resulted from oxidative biotransformation.

**Conclusions:**

Copanlisib was eliminated predominantly in the feces compared to urine as well as by hepatic biotransformation, suggesting that the clearance of copanlisib would more likely be affected by hepatic impairment than by renal dysfunction. The dual mode of elimination via unchanged excretion of copanlisib and oxidative metabolism decreases the risk of clinically relevant PK-related drug–drug interactions.

## Introduction

Phosphatidylinositol 3-kinase (PI3K) is a valuable target for clinical treatment of various types of cancer, as its biological activity is crucial for the translation of extracellular stimulation into intracellular signaling pathway, including cell growth and survival. Mutation of genes encoding PI3K and the related phosphatase PTEN (responsible for reversal of PI3K phosphorylation) are among the most frequently observed alterations in solid tumors [1–3]. Therefore a number of auspicious PI3K inhibitor candidate drugs including copanlisib are currently under clinical evaluation for treatment of a variety of solid tumors and blood cancers [4].

Copanlisib (BAY 80-6946; Bayer Pharma AG, Berlin, Germany) is a novel, intravenous, pan-class I phosphatidylinositol-3-kinase (PI3K inhibitor) with predominant PI3K-α and PI3K-δ inhibitory activity [5, 6], which are expressed in malignant B cells. Copanlisib has been shown to induce tumor cell death including apoptosis and inhibit proliferation of primary malignant B cell lines. In addition, copanlisib inhibited tumor growth in malignant B cell preclinical xenograft tumor models [7].

A dose escalation study has investigated general safety of copanlisib in the range from 0.1 to 1.2 mg/kg, defining 0.8 mg/kg as the maximum tolerated dose. At the MTD dose of 0.8 mg/kg (approximately equivalent to 60 mg absolute dose), copanlisib as monotherapy demonstrated promising efficacy in patients with solid tumors and hematological malignancies [8].

Copanlisib demonstrated dose proportional increases in maximum concentration (*C*_max_) and AUC from time zero to 25-h (AUC (0–25)) in the dose range 0.1–1.2 mg/kg (5–93 mg total dose). Copanlisib has a large volume of distribution 871 L (%CV 47.4), a reversible-free fraction plasma protein binding of 15.8% and a terminal phase half-life (*t*_1/2_) of 39.1-h (%CV 40.8) at the MTD dose of 0.8 mg/kg. The mean systemic plasma clearance is 18.9 (L/h) (%CV 51.2). No accumulation and no time dependency were observed after once weekly dosing when comparing PK on Cycle 1 Days 1 and 15 and Cycle 3 Day 15.

Population PK analysis showed no correlation between body weight, body surface area, or other body size-related factors and copanlisib clearance, indicating that body weight-based dosing does not reduce between-subject variability in copanlisib PK. Data from this analysis along with the exposure–response analysis for safety/efficacy was the basis to support the switch from a body weight-based dosing to a flat-dose regimen of copanlisib. The flat dose of 60 mg copanlisib infusion is used in all ongoing or planned clinical studies in adults [9]. A copanlisib dose of 12 mg was expected to be well tolerated in healthy subjects and the data derived from this dose will be predictive of results at higher doses given that the PK of copanlisib is linear up to and beyond the MTD dose (0.8 mg/kg which is equivalent to 60 mg).

This study was designed to elucidate the pharmacokinetics, especially focusing on metabolism and excretion pathways, of copanlisib after intravenous (i.v.) infusion in healthy volunteers to establish the safety features of the compound for further clinical evaluation.

## Materials and methods

### Study design and ethical approval

A single-center, open-label, non-randomized study in healthy male volunteers was performed to study pharmacokinetics, especially metabolism and excretion pattern, mass balance, as well as safety and tolerability after single 1-h i.v. administration of about 12 mg copanlisib containing about 2.76 MBq [^14^C]copanlisib. All subjects gave written informed consent; the study was conducted at PRA Health Sciences (Zuidlaren, The Netherlands) in accordance with the Declaration of Helsinki and was approved by an accredited institutional ethics committee (Medisch Ethische Toetsings Commissie, Assen, The Netherlands).

### Study population

Adult healthy male volunteers aged 45–55 years, with body weight greater or equal to 60 kg and body mass index (BMI) above or equal to 18 and below or equal to 30 kg/m^2^ were eligible for this study. Exclusion criteria included pre-existing diseases with assumed influence on study drug pharmacokinetics, history of hypersensitivity, severe allergies or diabetes, acute diseases, regular daily consumption of more than 1 L of methylxanthine-containing beverages, the inability to abstain from alcohol and methylxanthine-containing beverages or food (coffee, tea, cola, chocolate), drug/alcohol abuse or use of drugs with known strong interaction with cytochrome P-450 (CYP) 3A4, which is known to be involved in the metabolism of copanlisib in humans. In addition, smoking should have been stopped at least 60 days before study.

### Radiolabeled copanlisib and reference compound

**Fig. 1:**
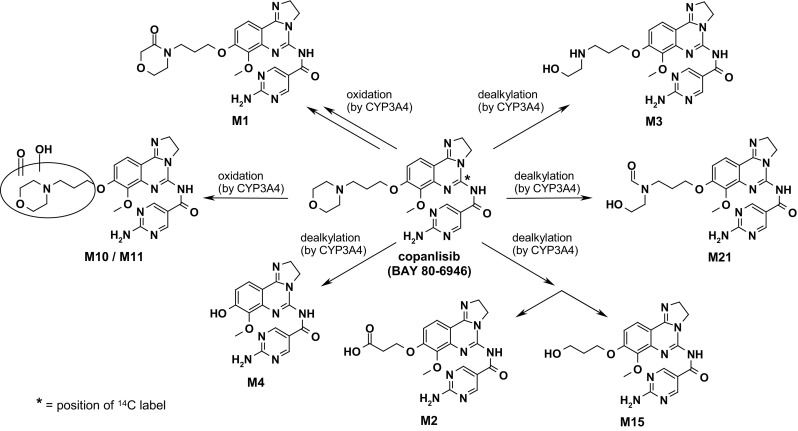
Chemical structure of copanlisib and its metabolites identified in human plasma, urine and feces following a single dose of 12 mg copanlisib (containing 2.76 MBq of [^14^C]-labeled copanlisib) as 1-h i.v. infusion in 6 healthy male volunteers

Introduction of ^14^C in position 2 of the quinazoline ring was performed to obtain [^14^C]-labeled copanlisib (Bayer AG, Wuppertal, Germany). Moreover, the human metabolite in plasma and urine, M1 (for chemical structure cf. Fig. 1) reference compound was synthesized in-house. Based on the data from animal studies and clinical trials, the calculated effective radiation burden after a single IV radioactivity dose of 2.76 MBq (75 µCI) ^14^C-radiolabeled copanlisib was approximately 0.8 mSv, falling within the International Commission on Radiological Protection 1992 Guidelines for Category 2a studies (0.1–1 mSv). The 12 mg dose was chosen because it was expected to be safe to be used in healthy volunteers corresponding to one of the lowest doses in the first-in-man study [10].

### Study conduct

The six healthy male volunteers received a single i.v. infusion of 12 mg copanlisib (2.76 MBq [^14^C]copanlisib) in a total volume of 40 mL as a 1-h infusion in the morning, 1-h after a light breakfast. Subjects remained fasting until 2-h after the end of the infusion. Volunteers stayed in-house until 14 days after the application and subsequently urine and feces were collected for 24-h on Day 16, 20, 27 and 34 if necessary, cf. below.

### Sample collection

Blood and plasma samples for pharmacokinetic evaluation and metabolite profiling were collected pre-dose, as well as 0.5, 1 (end of infusion), 1.5, 2, 3, 4, 6, 8, 10, 16, 24, 36, 48, 72, 96, 120, 144, 168, 192, 216, and 240-h after the start of the copanlisib infusion. Urine was collected pre-dose, at 0–12-h and 12–24 h after the start of the infusion and thereafter over 24-h intervals on Day 2 to Day 14 as well as on Day 16, 20, 27 and 34 (±1 day). Cumulative 24-h fecal samples were collected from Day 1 (day of infusion) to Day 14 and as well as on Day 16, 20, 27 and 34 (±1 day). Because the criteria for study release for all subjects have not been met at Day 14 (sum of excreted radioactivity in urine and feces less than 1% on two consecutive days), subjects were discharged and requested to collect urine and/or feces at home during specified 24-h intervals and bring these specimens to the clinic at specified days. The excreted radioactivity between ambulant visits during the prolongation phase of the clinical study was calculated using interpolation to get a more realistic total excretion value of all subjects (see "Pharmacokinetic analysis").

### Bioanalytics

#### Blood, plasma: mass spectrometry

All samples were frozen at −70 °C and analyzed within 60 days of sample collection. Stability of copanlisib and M1 had been established under these storage conditions for 1 year or more. High-performance liquid chromatography (HPLC) with tandem mass spectrometry (LC–MS/MS) was employed for determination of copanlisib and M1 in plasma and blood, the method had been fully validated according to current scientific and regulatory recommendations. The calibration ranges of the method for copanlisib and M1 were 0.5–250 and 0.5–95 ng/mL, respectively. Accuracy and precision of the method were 1.9–15.0 and 93.8–99.7% for copanlisib and 2.6–17.6 and 94.1–97.8% for metabolite M1.

#### Blood, plasma, urine, feces: radioactivity

After pooling and homogenization of urine and feces samples per time interval, aliquots were stored at −20 °C for total radioactivity determination and at −70°C for metabolic profiling (including blood/plasma samples). For determination of total radioactivity, samples were analyzed with a Perkin Elmer Tri-Carb™ 3100 TR liquid scintillation analyzer (Perkin Elmer, Waltham, MA, USA) with quenching correction. For metabolite profiling, plasma samples were treated with a mixture of acetonitrile (ACN) +0.2% acetic acid, after shaking and centrifugation, the supernatant was concentrated. Urine and feces samples were treated with solid-phase extraction (Waters HLB 30 mg, 1 mL, Waters, Milford, MA, USA) and liquid–liquid extraction (ACN + 0.2% trifluoroacetic acid/water, 80:20 volume/volume), respectively. Subsequently samples from plasma, urine and feces were analyzed by HPLC-LSC on Synergi Polar 4 µm 150 × 2 mm (Phenomenex, Aschaffenburg, Germany) with off-line radioactivity detection by Microbeta^2^™ Plus liquid scintillation counter with Ultima Flo™ AP scintillation fluid (Packard Instrument BV, Groningen, The Netherlands).

#### Chemical structure elucidation

Metabolite patterns in plasma, urine and feces were studied by the HPLC method with off-line radioactivity detection described above. Confirmation of chemical identity of copanlisib and its metabolites was generally performed employing reference compounds (copanlisib and M1), by comparison of HPLC retention times as well as by LC–MS/MS fragmentation data.

### Pharmacokinetic analysis

Pharmacokinetic parameters for copanlisib and M1 in plasma were calculated from LC–MS/MS data. For blood/plasma ratio determination, total radioactivity in urine and feces well as data from HPLC analysis data from radioactivity measurement by liquid scintillation counting (LSC) were considered. The amount of radioactivity recovered in the excreta (*A*_E_) was related to the administered radioactive dose to establish the radioactive balance. Arithmetic means and coefficients of variation (CV) were calculated for these percentages. Urine and feces were collected completely up to day 14 after administration and thereafter on four ambulatory visits [Day 16 (*N* = 6), Day 20 (*N* = 6), 27 (*N* = 5), 34 (*N* = 3)]. Cumulative excretion was calculated as sum of the collection intervals. The elimination of the total radioactivity in urine and feces for time-points beyond day 14 was performed by log-linear extrapolation between analyzed time-points. Amounts of radioactivity excreted in urine and feces between two ambulant visits were calculated per interpolated day by linear interpolation of log-transformed excretion data separately per matrix (ln*A*_E_(interpolated day) = Δdays × [ln*A*_E_(Visit *i* + 1) − ln*A*_E_(Visit *i*)]/[day (Visit *i* + 1) − day (Visit *i*)] + ln*A*_E_(Visit *i*), with *A*_E_ given as amount of radioactivity excreted in matrix per day and Δdays being the difference between the calculated day and the last available visit with *A*_E_ > 0). In case no feces sample was provided on an ambulant visit, this visit was neglected for interpolation. A similar approach has been described earlier [11]. The concentration/amount for each component of each sample/time-point was calculated by multiplying the total radioactivity concentration/amount of each sample with %-area of each component obtained after off-line HPLC separation. The amount of radioactivity in urine and feces at late time-points where no component was detected was distributed in the same percentage to the component (s) present at the time-point before.

Compartment-free analysis was performed employing WinNonlin software (version 5.3, Pharsight Certara, Princton, NJ, USA). From the concentration–time data for copanlisib, the maximum plasma concentration (*C*_max_), time to *C*_max_ (*t*_max_), area under the plasma concentration–time curve from time zero (start of infusion) to 24 h (AUC (0–24)) or to the time of the last quantifiable concentration (AUC (0–*t*_last_) as well as the AUC from time zero extrapolated to infinity (AUC (0–∞)), total body clearance (CL), volume of distribution (*V*_z_), terminal half-life (*t*_½_), as well as the mean residence time (MRT) were calculated. For the metabolite quantifiable in plasma, M1, the following parameters were calculated: AUC (0–*t*_last_), *C*_max_, as well as *t*_max_.

### Statistical evaluation

Pharmacokinetic parameters were evaluated by descriptive data analysis without formal hypothesis testing by calculating either medians (*t*_max_, *t*_last_) or geometric means and %CV (remaining parameters). Excretion data (urine, feces, total) were evaluated by arithmetic means.

### Safety assessment

Physical examination, blood pressure, pulse rate, body temperature and body weight, laboratory parameters and 12-lead electrocardiogram (ECG), as well as adverse events using the common terminology criteria for adverse events (CTCAE) Version 4.03, were monitored throughout the study for safety assessment.

## Results

### Subject demographics, disposition, and safety

Initially 18 subjects were screened, of which 6 were finally dosed in line with the study protocol. All 6 were males, 5 Caucasians, 1 Asian with an age of 54.8 ± 5.2 years (arithmetic mean ± standard deviation, range 47–61 years) and a body mass index of 24.9 ± 2.2 kg/m^2^. All were non-smokers and reported at the most light alcohol use. No relevant protocol deviations were noted.

No death or SAEs were reported. Three out of the 6 volunteers experienced at least one treatment emergent adverse event (TEAE). A total of 5 gastrointestinal TEAEs (MedDRA preferred terms: abdominal pain, nausea, flatulence, frequent bowel movements and diarrhea) as well as one event each of myalgia and oropharyngeal pain, respectively. The intensity of these TEAEs was generally mild-to-moderate (CTCAE grade 1 or 2), except one case of grade 3 abdominal pain occurring in one patient 8 days after the copanlisib administration, which was not considered drug-related by the investigator and resolved within 24-h. In total, 2 subjects experienced TEAEs, which were assessed as being related to the study drug copanlisib, including abdominal pain, diarrhea and nausea. A transient decrease in neutrophils and leukocytes was observed in all 6 healthy volunteers, but all were above 2/3 of the lower limit for normal values. No clinically relevant changes in other laboratory values, ECG or vital signs were observed.

### Pharmacokinetics

#### Blood, plasma

**Fig. 2:**
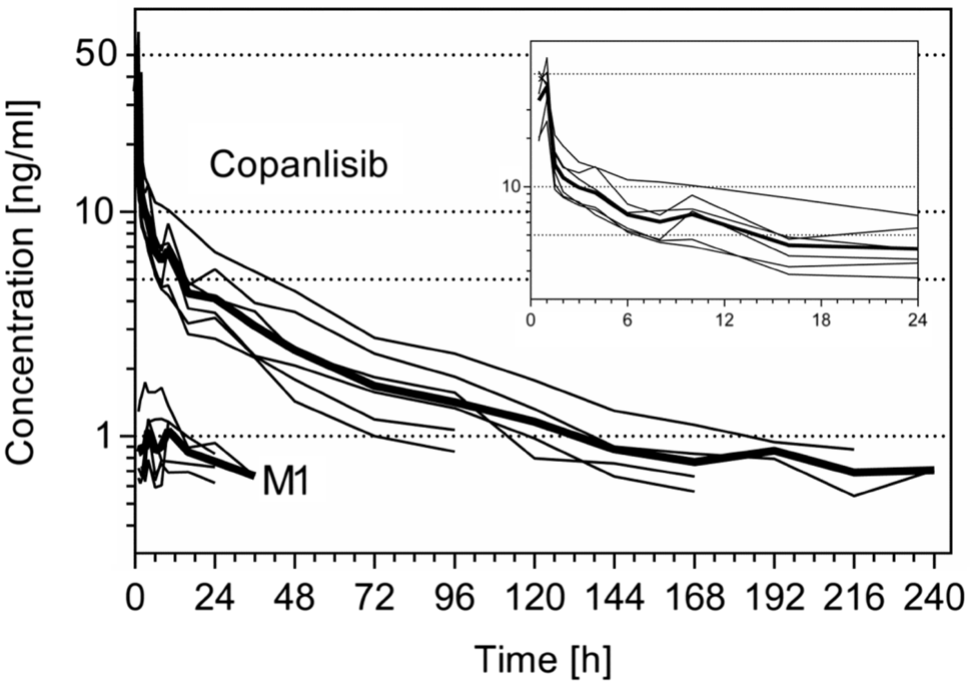
Plasma concentration–time profiles of copanlisib (LC–MS/MS data) and metabolite M1 (individual *solid line*, geometric mean *thick line*) following a single dose of 12 mg copanlisib (containing 2.76 MBq of [^14^C]-labeled copanlisib) as 1-h i.v. infusion in 6 healthy male volunteers (*insert* copanlisib profiles for the first 24-h)

The pharmacokinetic parameters for copanlisib and metabolite M1 in plasma are given in Table 1. Maximum plasma concentrations of 44.1 ng/mL (geometric mean, %CV 33.6%) were observed mostly at the end of infusion, followed by a rapid decline in the plasma concentration–time profile of copanlisib within the first 30 min after ending the infusion and a slower decline afterwards (Fig. 2). The lower limit of the quantification of the analytical method (i.e., 0.5 ng/mL) was reached 96–240-h after start of infusion. The mean terminal half-life was 52.1-h (range 40.4–67.5-h). Volume of distribution was high with about 1870 L (%CV 24.8%, range 1250–2450 L) and total body clearance was low with about 24.8 L/h (%CV 41.3%, range 14.2–40.8 L/h).

**Table 1:** Pharmacokinetic parameters of copanlisib and its major metabolite M1 in plasma following single-dose administration of 12 mg copanlisib (containing 2.76 MBq of [^14^C]-labeled copanlisib) as 1-h infusion in 6 healthy male volunteers

	Unit	Geometric mean (%CV)	Range
Copanlisib			
AUC (0–∞)	ng·h/mL	442 (41.2%)	269–769
AUC (0–*t*_last_ *)*	ng·h/mL	392 (46.4%)	223–710
AUC (0–24)	ng·h/mL	176 (30.7%)	121–273
*C*_max_	ng/mL	44.1 (33.6%)	25.6–63.4
*t*_max_^a^	h	1	0.5–1
CL	L/h	24.8 (41.3%)	14.2–40.8
*V*_z_	L	1870 (24.8%)	1250–2450
*t*_½_	h	52.1 (20.9%)	40.4–67.5
MRT	h	62.7 (22.3%)	47.7–80.3
*t*_last_^a^	h	168	96–240
M1			
AUC (0–*t*_last_ *)*	ng·h/mL	12.4 (99.7%)	4.36–37.8
*C*_max_	ng/mL	1.16 (25.9%)	0.783–1.74
*t*_max_^a^	h	6	3–10
*t*_last_^a^	h	24	8–36

AUC (0–∞) area under the concentration–time curve from start of infusion extrapolated to infinity, AUC (0–t_last_) area under the concentration–time curve from start of infusion to time of last quantifiable concentration, AUC (0–24) area under the concentration–time curve for the first 24-h from start of infusion, *C*_max_ maximum concentration, *t*_max_ time to maximum concentration, CL total body clearance, *V*_z_ volume of distribution during terminal phase, *t*_½_ terminal half-life, MRT mean residence time, *t*_last_ time of last quantifiable concentration

^a^Median

**Fig. 3:**
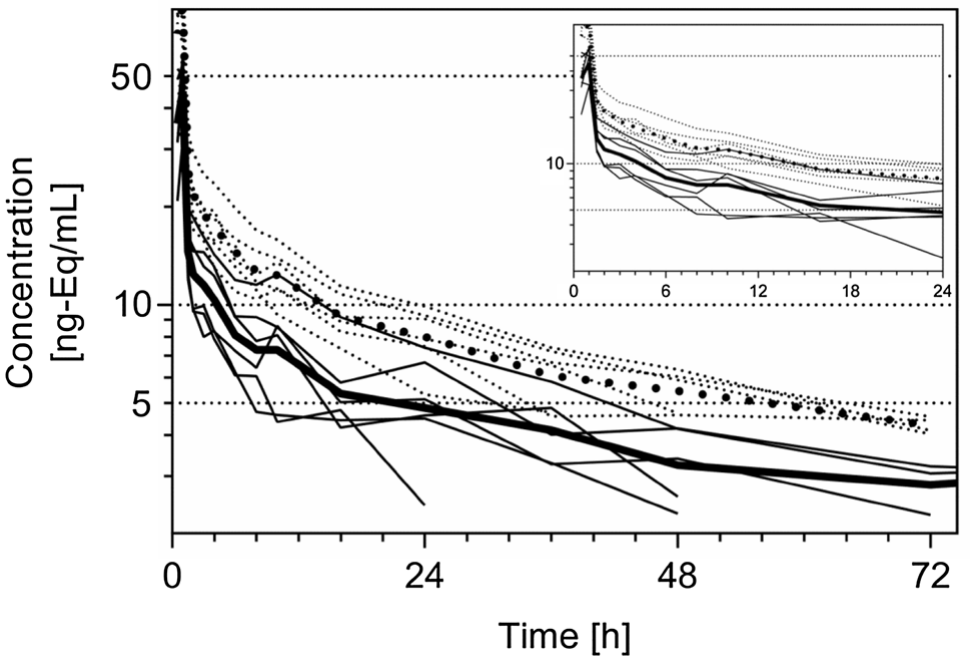
Individual (*thin line*) and geometric mean (*thick line*) plasma (*sold lines*) and blood (*dotted*) concentration–time profiles of total radioactivity following a single dose of 12 mg copanlisib (containing 2.76 MBq of [^14^C]-labeled copanlisib) as 1-h i.v. infusion in 6 healthy male volunteers

Concentrations of radioactivity in whole blood were consistently higher than in plasma (Table 2), while no differences were observed in the elimination from these two compartments (Fig. 3) and results were in good accordance to data for copanlisib in plasma obtained from LC–MS/MS analytics described above (Table 1). Moreover, the blood-to-plasma ratio of total radioactivity hardly exceeded 2 for both, *C*_max_ as well as AUC, indicating no excessive accumulation of drug-related material in blood cells. Results from HPLC with off-line radioactivity detection confirmed the identity of most of the radioactivity in the central circulation as copanlisib (data not shown). Radioactivity was extractable from plasma with high recovery (typically >90%), giving no indication of covalent adduct formation via chemically reactive metabolites.

**Table 2:** Pharmacokinetic parameters of radioactivity in plasma and whole blood following single-dose administration of 12 mg copanlisib (containing 2.76 MBq of [^14^C]-labeled copanlisib) as 1-h infusion in 6 healthy male volunteers

	Unit	Geometric mean (% CV)	Range
Plasma			
AUC (0–*t*_last_)	ng-Eq·h/mL	324 (54.1%)	150–601
*C*_max_	ng-Eq/mL	45.6 (26.0%)	32.1–57.6
*t*_max_^a^	h	1	0.5–1
*t*_last_^a^	h	60	24–96
Blood			
AUC (0–*t*_last_)	ng-Eq·h/mL	558 (28.8%)	347–756
*C*_max_	ng-Eq/mL	83.5 (26.3%)	59.2–114
*t*_max_^a^	h	1	0.5–1
*t*_last_^a^	h	72	36–72
Ratio blood:plasma			
AUC (0–*t*_last_)		1.71 (33.5%)	1.12–2.44
*C*_max_		1.83 (15.2%)	1.52–2.21

CV% geometric coefficient of variation, AUC (0–*t*_last_) area under the concentration–time curve from start of infusion to time of last quantifiable concentration, *C*_max_ maximum concentration, *t*_max_ time to maximum concentration, *t*_last_ time of last quantifiable concentration

^a^Median (range)

#### Urine, feces

**Fig. 4:**
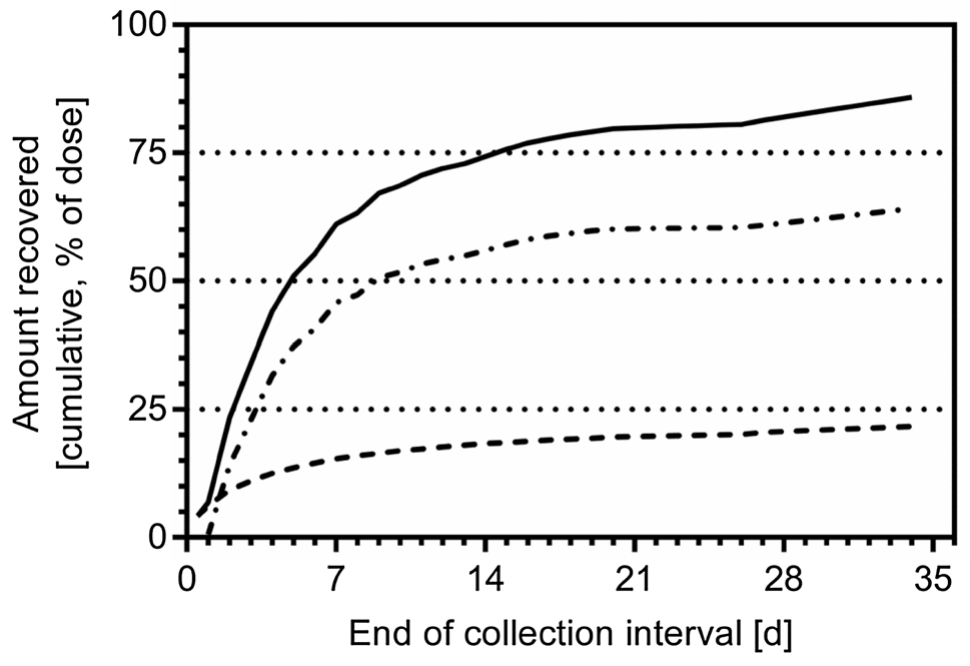
Arithmetic mean cumulative excretion of radioactivity in urine (*dashed line*), feces (*lines with black circle*) as well as sum of urine and feces (*thin line*) following a single dose of 12 mg copanlisib (containing 2.76 MBq of [^14^C]-labeled copanlisib) as 1-h i.v. infusion in 6 healthy male volunteers

**Fig. 5:**
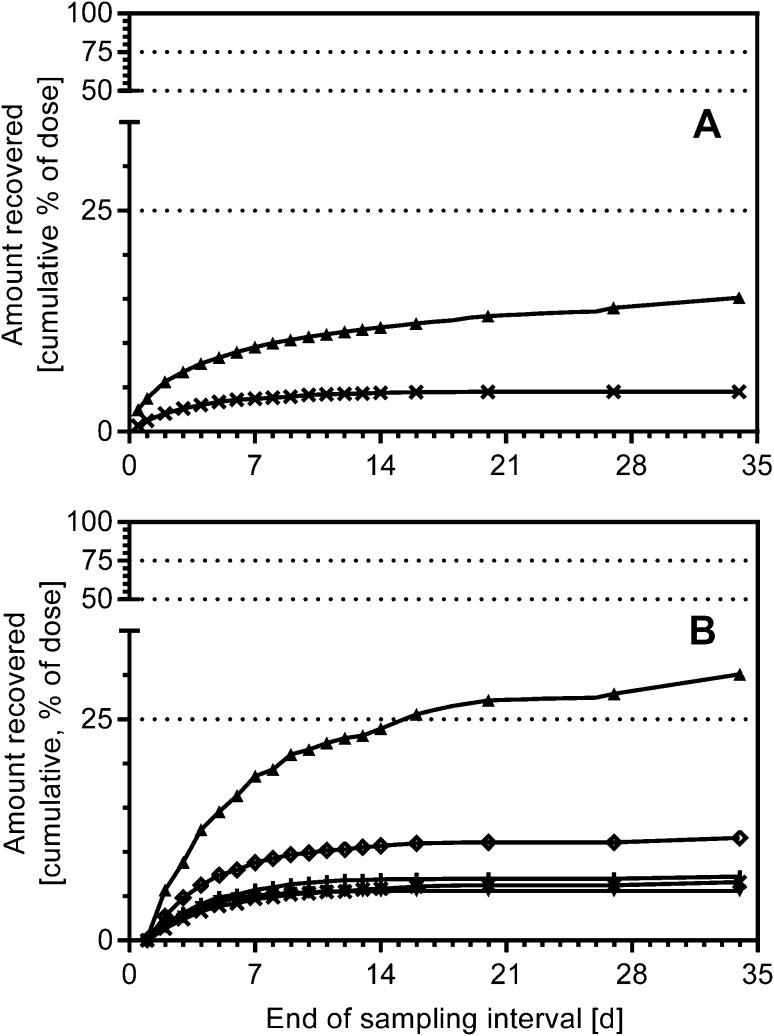
Arithmetic mean cumulative excretion of copanlisib (*filled up triangle*) and metabolites M1 (Χ), M2 (*diamond*), M3 (*vertical line*) and M4 (*filled down triangle*) in urine (**a**) and feces (**b**) following single-dose administration of 12 mg copanlisib (containing 2.76 MBq of [^14^C]-labeled copanlisib) as 1-h i.v. infusion in 6 healthy male volunteers (interpolation of excretion between late-phase isolated sampling days)

Because the criteria for study release for all subjects have not been met at Day 14 (sum of excreted radioactivity in urine and feces less than 1% on two consecutive days), subjects were discharged and requested to collect urine and/or feces at home during specified 24-hour intervals (Day 16, 20, 27, and 34) and bring these specimens to the clinic at specified days. The excreted radioactivity between ambulant visits during the prolongation phase of the clinical study was calculated using interpolation to get a realistic total excretion value of all subjects. One subject was released after Day 20, 2 subjects were released after Day 27, while 3 subjects provided excretion data until Day 34. Based on study release at Day 20 (*N* = 6), Day 27 (*N* = 5) and Day 34 (*N* = 3), the excreted total radioactivity in urine and feces amounted to 81.0 ± 2.1% (range 77.8–83.0%), 84.9 ± 2.3% (range 82.3–88.5%), and 88.3 ± 2.9% (range 85.2–91.0%), respectively. In total, mean total radioactivity recovery amounted to 85.9 ± 3.4% (range 81.7–91.0%) based on all 6 subjects after individual study release (Day 20–Day 34). Based on study release at Day 20 (*N* = 6), Day 27 (*N* = 5) and Day 34 (*N* = 3), the excreted radioactivity in feces amounted to 61.0 ± 4.1% (range 54.8–66.7%), 63.4 ± 4.5% (range 56.7–67.4%), and 66.1 ± 3.2% (range 62.4–68.1%), respectively, and in urine to 20.0 ± 3.5% (range 16.3–26.5%), 21.5 ± 3.9% (range 17.0–27.4%), and 22.2 ± 1.4% (range 20.6–23.1%), respectively. In total, 64.2 ± 4.5% (range 56.7–68.1%) of the radioactive dose was excreted in feces as major route and 21.7 ± 3.6% (range 17.0–27.4%) in urine as minor route of excretion based on all 6 subjects after individual study release (Day 20–Day 34) (Fig. 4). Copanlisib was the predominant component excreted in both, urine and feces with approximately 15 and 30% of the dose recovered, respectively (Fig. 5). Excretion was slow for both, urinary as well as fecal route of elimination, known for drugs with a long half-life [12]. Overall, 45.1% of the dose were recovered as unchanged copanlisib (range 33.3–53.8%) and 40.8% of dose as metabolites (27.3–54.4%).

### Metabolite profile

Comparing AUC (0–*t*_last_) of copanlisib obtained by LC–MS/MS analysis with those of AUC (0–*t*_last_) of total radioactivity for the same interval (where *t*_last_ is defined by the time-point with detectable radioactivity), revealed that about 84% (arithmetic mean for all six subjects, range 74–91%) of total radioactivity AUC (0–*t*_last_) was covered by copanlisib (Table 3; Fig. 2). LC–MS/MS analysis also revealed the presence of metabolite M1 in all subjects and PK analysis revealed that about 5% (arithmetic mean for all six subjects, range 1.6–12%) of total radioactivity AUC (0–*t*_last_) was covered by M1 (Table 3; Fig. 2). Together with the analysis of the urine samples where about 90% of the radioactivity is covered by copanlisib and its M1, it is suggested that total AUC in human plasma is almost solely covered by copanlisib and metabolite M1 and consequently no unknown major plasma metabolite was present. Details on the excretion profile of copanlisib and metabolites are provided in Table 4. In total, about 31% of the dose administered was recovered in feces as the sum of metabolites M1, M2, M3 and M4. In urine M1 was the only relevant metabolite (4.5% of dose), while the dominant metabolite found in fecal excretion was M2, the aliphatic carbonic acid resulting from oxidative dealkylation at the aliphatic morpholine spacer side-chain.

**Table 3:** Comparison of AUC (0–*t*_last_) of total radioactivity with copanlisib and metabolite M1 following single-dose administration of 12 mg copanlisib (containing 2.76 MBq of [^14^C]-labeled copanlisib) as 1-h infusion in 6 healthy male volunteers

Total radioactivity	Copanlisib (LC–MS)		M1 (LC–MS)		Range of *t*_last_ (h)
Range of AUC (0–*t*_last_) (ng-Eq·h/mL)	Range of AUC (0–*t*_last_) of total radioactivity (ng-Eq·h/mL)	Range of AUC (0–*t*_last_) of total radioactivity (%)	Range of AUC (0–*t*_last_) (ng-Eq·h/mL)	Range of AUC (0–*t*_last_) of total radioactivity (%)	
150–601	212–549	73.8–91.3	4.36–37.8	1.6–12.2	24–96
		Average 84.4		Average 4.88

**Table 4:** Excretion pattern of copanlisib and its metabolites in urine and feces (arithmetic mean, range) following single-dose administration of 12 mg copanlisib (containing 2.76 MBq of [^14^C]-labeled copanlisib) as 1-h infusion in 6 healthy male volunteers

Matrix	Amount excreted in (% of dose)	
	Urine	Feces
Total amount excreted	21.7 (17.0–27.4)	64.2 (56.7–68.1)
Parent compound		
Copanlisib	15.0 (9.52–18.7)	30.1 (16.8–37.9)
Metabolites		
M1	4.47 (2.53–6.79)	6.39 (4.34–8.66)
M2	0.34 (0.10–0.52)	11.8 (9.91–13.0)
M3	0.32 (0–0.62)	7.32 (5.61–11.6)
M4	0.15 (0.04–0.32)	5.61 (1.74–9.53)
M10/M21	0.14 (0–0.41)	1.84 (0.52–3.06)
M11/M15	0.13 (0–0.47)	0.62 (0.20–1.48)

Interestingly, the amounts of M1 excreted via urine and feces were 4.5 and 6.6%, respectively (Fig. 5), and in total 11.6% of the dose were recovered as M2 from feces, i.e., about the same amount as for the combined excretion of M1 in both urine and feces (11.1%). Products of further oxidative degradation of the morpholine moiety (M3) as well as oxidative degradation of the alkoxy spacer for the morpholine ring (M4) accounted for 7.2% and 5.6%, respectively, and were found at relevant amounts only in feces.

Over 98% of the radioactivity excreted could be assigned to known structures, including some minor intermediates/products of oxidative metabolism of the alkoxy morpholine side-chain (M10, M11, M15, M21), each representing <2% of dose applied (Fig. 1). The ratio of excretion as unchanged copanlisib to metabolites ranged from 60:40 (*N* = 3) over 50:50 (*N* = 2) to 32:68 (*N* = 1), no correlation with total clearance was observed in the small sample size included in this trial.

## Discussion

Single i.v doses of 12 mg copanlisib containing 2.76 MBq [^14^C] copanlisib administered to 6 healthy subjects were well tolerated without any serious adverse event at the dose tested. Three mild-to-moderate gastrointestinal adverse events related to copanlisib were reported, which are well-known effects of PI3K inhibitors [8].

Copanlisib was rapidly and extensively distributed throughout the body, with a large volume of distribution of 1870 L. Total body clearance of copanlisib was low (24.8 L/h). The mean terminal half-life was 52-h (40–67-h) and is in accordance with the known PK of copanlisib (range 16.9–72.7-h) [8]. The mean recovery of total radioactivity was about 86% within a collection interval of 20–34 days: about 64% in feces and about 22% in urine. Unchanged copanlisib was the main component excreted in both, urine and feces with approximately 15 and 30% of the dose recovered, respectively.

Besides a high fraction of parent compound excreted unchanged into urine and feces (45.1%), metabolites M1 and M2 were the predominant species excreted in urine and feces, respectively. The chemical structures of these metabolites as well as the extraction properties from biological samples observed in this trial do not indicate potential for any covalent adduct formation or other signs for high chemical reactivity. From in vitro studies on hepatic metabolism in man as well as in toxicologically relevant animal species, it is known that copanlisib is mostly converted via oxidation of the morpholine moiety (M1, M10, M11) with subsequent degradation of this ring structure, resulting in structures such as M3 and M21. Besides these reactions, oxidative degradation of the alkoxy side-chain used as the spacer for the morpholine moiety were observed in vitro, such as M2, M4 or M15. This spectrum of metabolites has been confirmed in vivo in man by the results of this trial. These metabolites are in line with the established biotransformation products of a morpholine moiety in several other compounds, describing formation of lactam and lactone metabolites as the predominant pathways, with further degradation of the ring structure by oxidation [13]. The removal of the morpholine ring structure, as observed for the formation of metabolite M2, is well-established for mammalian biotransformation of related alkylmorpholine-structures [14, 15], although sometimes without the extensive oxidation of the remaining alkoxy-chain observed for copanlisib, leading to M2 and finally resulting in M4. However, apparently there are significant differences between species and between in vitro and in vivo findings regarding the extent of oxidative degradation of morpholine structures, as described, e.g., for gefitinib, which undergoes oxidation of the alkoxy side-chain only in vitro, while in vivo in man only oxidation products of the morpholine with ring opening are described [16–18].

Other drugs containing morpholine structures, such as reboxetine or indeloxazine, have been found to be stable after initial morpholine oxidation, not undergoing ring opening and degradation as found for copanlisib, resulting in M3 and M21. Linkage of morpholine via ring-carbon (not via the nitrogen, as, e.g., in copanlisib) might be the structural prerequisite for this increased oxidative stability [19, 20]. Interestingly, even two methyl groups vicinal to the morpholine oxygen as in sonidegib or other drugs cannot completely abolish oxidative morpholine degradation, although metabolic turnover is diminished significantly by this dimethyl-morpholine structure [21, 22]. The major metabolites observed are in line with the established knowledge on the oxidative metabolism of morpholine-containing drugs.

Morpholine *N*-oxides often described as relevant metabolites of related drugs in mammalians [14, 15] were not found at significant amounts for copanlisib. Moreover, rarely observed quaternary nitrogen glucuronides observed for other morpholine-containing structures were also not found to any relevant extent [23].

The only metabolite observed in the systemic circulation at relevant concentrations was M1, at about 5% of the exposure of total radioactivity AUC, exhibiting affinity to the clinical target in the same order of magnitude as found for the parent compound (Bayer AG, data on file). Therefore, metabolites can be considered to be of limited relevance for the clinical efficacy of copanlisib. From data obtained in bile-duct cannulated animals (Bayer AG, data on file), involvement of extrabiliary pathways of clearance, directly via the gut wall are considered highly probable. Moreover, alterations in metabolic clearance via intrinsic or extrinsic factors might modulate clinical efficacy or safety of copanlisib to limited amounts, as only about 41% of the dose were found to be cleared via metabolism. As in vitro data suggest predominant involvement of cytochrome P450 3A4 in the primary biotransformation step in the metabolism of copanlisib in humans (Bayer AG, data on file), currently the potential interaction with a clinically relevant inhibitor of this subtype, itraconazole, is studied in a clinical trial [24].

Comparing the renal clearance of copanlisib (*A*_e,ur_/AUC) with the unbound glomerular filtration rate of copanlisib (GFR × fraction unbound in plasma of 15.8% [6]) passive renal filtration of copanlisib accounts for about one to two-thirds of the observed renal clearance of copanlisib (i.e., about 15% of total body clearance), indicating likely involvement of active renal secretion. Based on in vitro data, copanlisib is a substrate for the efflux transporter *P*-glycoprotein (P-gp) and breast Cancer Resistance Protein (BCRP) (Bayer AG, data on file) and these transporters might contribute to overall excretion of unchanged copanlisib. Only about 22% of the dose was recovered from urine, therefore renal excretion does not appear to be a critical clearance pathway for both, parent compound as well as M1, the only relevant metabolite in urine. Additionally, both compounds undergo significant fecal excretion. Consequently, limitations in renal function should not be of crucial relevance for copanlisib pharmacokinetics.

In summary, a single dose of 12 mg [^14^C]copanlisib (2.76 MBq) in 6 healthy volunteers was well tolerated with about 86% of copanlisib-related radioactivity was based on the administered dose recovered in feces (about 64%) and urine (about 22%) within a collection interval of 20–34 days indicating that renal excretion is not a major route of elimination. Unchanged copanlisib was the main component excreted in both, urine and feces with approximately 15% and 30% of the dose recovered, respectively. The other half of copanlisib-related radioactivity was excreted as metabolites resulting from oxidative biotransformation. The dual mode of elimination, occurring via both excretion of parent compound and oxidative metabolism decreases the risk of PK-related drug–drug interaction.
